# Acute ST-Elevation Myocardial Infarction after Coronary Stent Fracture

**Published:** 2015-10-27

**Authors:** Abbasali Rafighdust, Ali Eshraghi

**Affiliations:** Imam Reza Hospital, Mashhad University of Medical Sciences, Mashhad, Iran.

**Keywords:** *Stents*, *Drug-eluting stents*, *Treatment failure*, *Myocardial infarction*

## Abstract

The invention of the drug-eluting stent (DES) has brought about revolutionary changes in the field of interventional cardiology. In the DES era, in-stent restenosis has declined but new issues such as stent thrombosis have emerged. One of the emerging paradigms in the DES era is stent fracture. There are reports about stent fracture leading to in-stent restenosis or stent thrombosis. Most of these reports concern the Sirolimus-eluting stent. The present case is a representation of a Biolimus-eluting stent fracture. We introduce a 64-year-old male patient, for whom the BioMatrix stent was deployed in the right coronary artery. Five months after the implantation, he experienced acute myocardial infarction, with stent fracture leading to stent thrombosis being the causative mechanism. Another DES (Cypher) was used to manage this situation, and the final result was good.

## Introduction

In the path of the evolution of interventional cardiology, in-stent restenosis was an obstacle which was overcome by the introduction of the drug-eluting stent (DES). Neointimal proliferation was the Achilles' heel of the bare-metal stent (BMS) and the causative mechanism of in-stent restenosis. The DES can halt the growth of the neointimal tissue and reduce restenosis.^1^ Nevertheless, the DES era has witnessed other challenges such as stent thrombosis, stent fracture, and aneurysm formation.^2^ Stent fracture is a relatively rare entity reported more frequently with the Sirolimus-eluting stent.^3^ Various risk factors for stent fracture have been noted such as stent length, lesion type, and stent type. New advances in image acquisition and processing such as StentBoost may enhance fracture detection.^4^

In this case report, we present a rare case of stent fracture culminating in acute ST-elevation myocardial infarction (MI) with the implantation of the Biolimus-eluting stent. 

## Case Report

Our patient was a 64-year-old man, who complained of frequent chest pain and had a positive exercise stress test. His only risk factor for coronary artery disease (apart from his age) was smoking history. On July 10^th^, 2011, the patient underwent coronary angiography (Siemens Zee, Artis, 2010), revealing an unremarkable left coronary tree but significant proximal stenosis in the right coronary artery (RCA). At the time, a BioMatrix stent (3.5 mm in diameter, 15 mm long) (Biosensors Europe SA) was deployed in the RCA and post dilation with a non-compliant balloon (3.5 mm in diameter, 15 mm long) (Sapphire, OrbusNeich Medical) was done. In our laboratory, this non-compliant balloon was the most suitable balloon on the shelf then. The final result was good, and the patient was discharged the day after the procedure with a dual antiplatelet regimen (Aspirin and Clopidogrel). 

**Figure 1:**
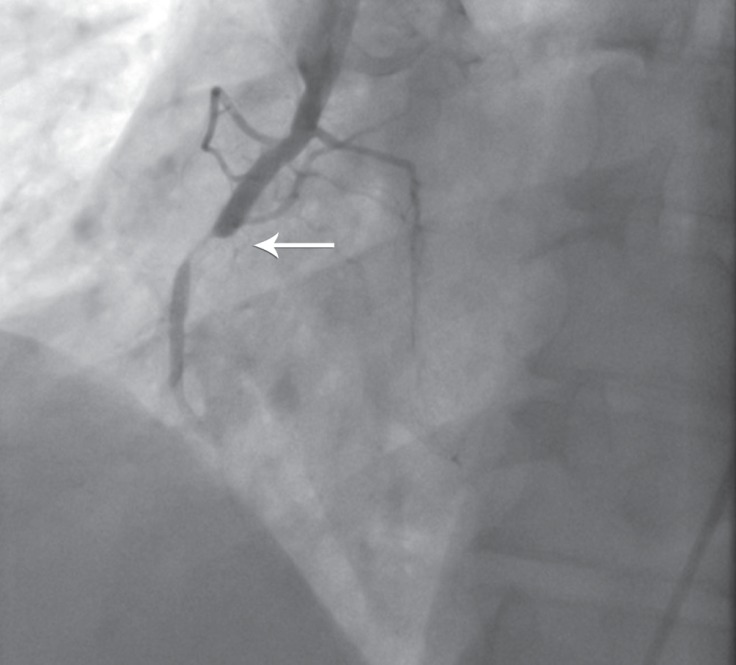
Total occlusion of right coronary artery in right anterior oblique view of coronary angiography (arrow)

**Figure 2:**
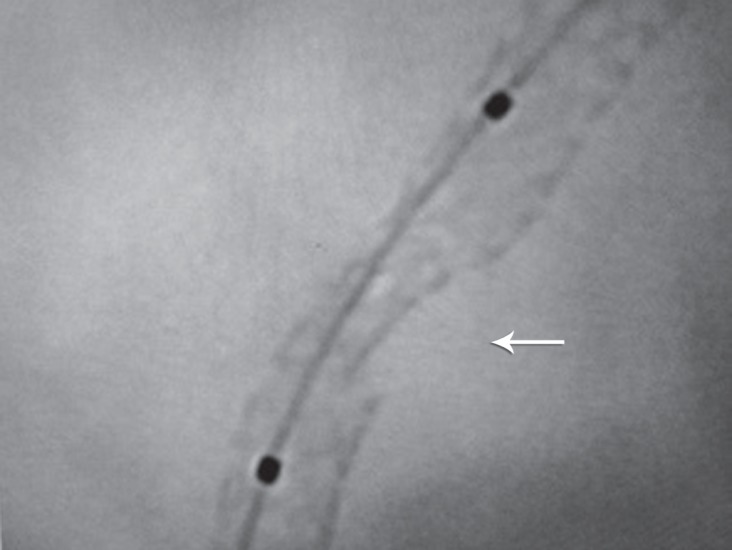
Stent enhancement clearly shows stent fracture (arrow) in right anterior oblique view of coronary angiography

About five months later, the patient experienced severe resting chest pain and after admission in the Emergency Department, his electrocardiogram (ECG) showed ST elevation in the inferior leads. Considering his medical history, stent thrombosis was the first diagnosis. The patient claimed that he had not stopped his medication. He was, therefore, transferred to the Catheterization Laboratory, where he underwent angiography. A high-grade lesion was detected in the middle of the RCA stent (Figure 1), and the stent fracture was documented with stent enhancement (StentBoost, Siemens Zee Artis) (Figure 2). After thorough explanation was given to the patient, the lesion was dilated with a Sapphire Balloon (2.5 mm in diameter, 15 mm long) and a Cypher stent (3.5 mm in diameter, 18 mm long) (Cordis Europe N.V.) was deployed in place of the fractured stent. Post dilatation was not performed, and stent inflation pressure was up to 18 atm. 

The final result was good, and the patient was discharged two days after the procedure. 

**Figure 3:**
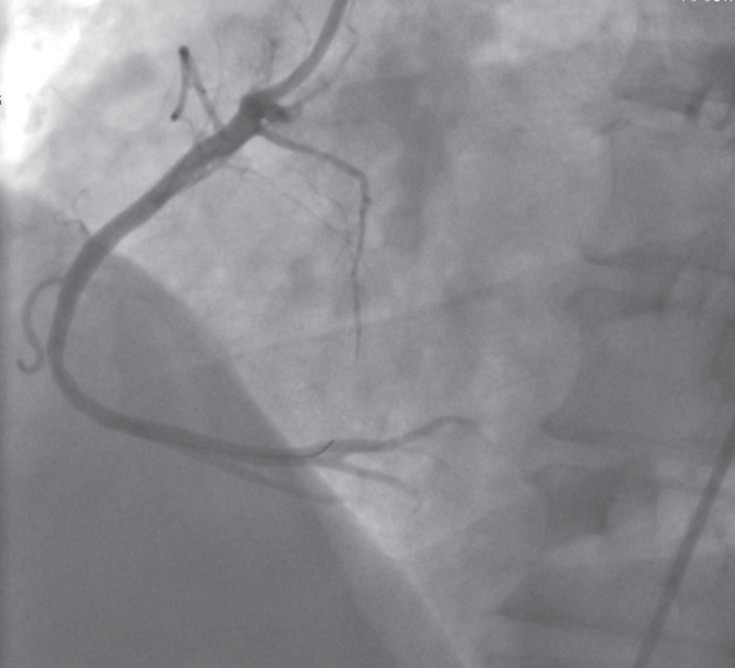
Finally, right anterior oblique angiography showed that there was no evidence of residual stenosis after stent deployment.

## Discussion

Stent fracture is a rare clinical entity and must be considered as one of the complications of the DES implantation. In the BMS era, in-stent restenosis was a more important issue and some were of the notion that the neointimal tissue might exert some protective effects against stent fracture. Indeed, in the BMS ear, stent fracture was relatively rare or unnoticed.^3^

Most cases of stent fracture are minor. The frequency of stent fracture has been reported to be within a range of 1% to 30%.^1^^, ^^5^^-^^7^ The DES fracture with little luminal narrowing may have a benign course and remain unnoticed. However, the DES fracture may lead to insufficient drug delivery and in-stent restenosis in addition to other consequences such as stent thrombosis, acute coronary syndromes, and even aneurysm formation.^8^ The predictors of stent fracture risk are longer stent length, stent overlap, angulated and calcified lesion, Sirolimus-eluting stents, RCA implantation, saphenous vein implantation, and aggressive expansion.^1^^, ^^8^^-^^11^ Most cases of coronary stent fractures are in the RCA and in Sirolimus-eluting stents. Mechanical factors are important in the DES fracture. It has been demonstrated that the DES straightens the vessels, which renders the stent exposed to elevated mechanical forces.^3^ This explains the higher incidence of the DES fracture in the RCA position. There is a suggestion that the higher reported incidence of the DES fracture in the Sirolimus-eluting stent is due to a more rigid platform of this type of the DES.^3^ There are also reports of the DES fracture in other second-generation types of the DES such as Zotarolimus-eluting, Paclitaxel-eluting, and Everolimus-eluting stents.^1^^, ^^2^

In our patient, the DES fracture occurred in the RCA and the RCA lesion was angulated. We performed high pressure post dilation after the DES implantation as a routine. It has been suggested that high-pressure stent deployment may be a risk factor for the DES fracture.^3^ Be that as it may, there is still a general consensus about high-pressure DES deployment. 

One interesting feature of the case presented here is that the DES utilized was a Biolimus-eluting stent (BioMatrix). Whereas there are case reports of stent fracture in the Nobori stent (another Biolimus-eluting stent), such reports are few and far between as regards the BioMatrix stent.^12^ The highest rates of stent fracture have been reported with the Cypher stent; however, there is no conclusive evidence that any type of the DES is superior to another when it comes to stent fracture. That is why we chose the Cypher stent in the second procedure.

## Conclusion

Stent fracture may be one of the etiologies of the DES failure and as such demands sufficient consideration.

## Notes:

***This paper should be cited as:*** Rafighdust AA, Eshraghi A. Acute ST-Elevation Myocardial Infarction after Coronary Stent Fracture. J Teh Univ Heart Ctr 2015;10(4):205-207.
